# Vaginal delivery is safely achieved in pregnancies complicated by spinal cord injury: a retrospective 25-year observational study of pregnancy outcomes in a national spinal injuries centre

**DOI:** 10.1186/s12884-020-2752-2

**Published:** 2020-01-29

**Authors:** Katherine Robertson, Rehana Dawood, Felicity Ashworth

**Affiliations:** Buckinghamshire NHS Trust, Stoke Mandeville Hospital, Mandeville Rd, Aylesbury, HP21 8AL UK

**Keywords:** Retrospective study, Spinal cord injury, Pregnancy, Pregnancy complications, High risk

## Abstract

**Background:**

Women with spinal cord injuries (SCI) represent a high risk population during pregnancy with comparatively few studies in the literature regarding their management and pregnancy outcomes, due to the relative rarity of the condition. Our objective was to assess pregnancy outcomes in women with spinal cord injury.

**Methods:**

We performed a retrospective observational study of pregnancy outcomes by reviewing maternity records of all pregnant women with SCI attending the National Spinal Injury Centre at Buckinghamshire NHS Trust between 1991 and 2016. The outcome measures were Maternal demographic data, antenatal complications, method of anaesthetic, intrapartum data (gestation at delivery, onset of labour, mode of delivery, indication for obstetric intervention) and neonatal outcomes (low birth weight, stillbirth, neonatal death).

**Results:**

Fifty women with a total of 68 pregnancies were identified. Five patients sustained SCI during pregnancy and the remaining 63 pregnancies were conceived at least 1 year after SCI, of which 45 pregnancies had a SCI at T10 or above (73%) and 23 pregnancies at T11 or below (27%). The most common antenatal complications in SCI patients were worsening of spasms (38%) and urinary tract infection (24%). Preterm delivery occurred in 18% of women. Vaginal delivery was achieved in 77% of pregnancies, including 14% instrumental delivery rate and 23% Caesarean delivery rate.

**Conclusions:**

Our findings support the current evidence that pregnancy outcomes are generally successful and that vaginal delivery can be safely achieved in the majority of women, independent of the level of SCI.

## Background

Spinal cord injury (SCI) affects over 40,000 people in the UK [1] and approximately 285,000 in the US [2]. Approximately 19% are female and over 50% of new injuries are in women between 16 and 30 years old, with one study reporting that 14% of women with SCI became pregnant after injury [3].

Spinal cord injury is considered high risk in pregnancy and presents unique challenges. Common medical complications include urinary tract infections, pressure ulcers, impaired pulmonary function, anaemia, venous thromboembolism and autonomic dysreflexia [4]. Obstetric complications include preterm labour, increased risk of Caesarean section and unattended delivery whilst anaesthetic complications with regional anaesthesia and the management of autonomic dysreflexia may also occur [5]. Consensus recommendations for the obstetric management of women with spinal cord injuries exist [6] however decisions about mode of delivery are complex and take into account the severity and type of injury, the patient’s preferences as well as the potential for complications.

The rarity of the condition means that most maternity units will encounter very few obstetric patients with SCI. There are relatively few observational studies describing pregnancy outcomes in this population, of which the largest case series of 175 women was reported in 1972 [7] and the two most recent in 2013 [8] and 2017 [9] both reporting the outcomes of 37 pregnancies in tertiary centres in Canada and France, describing successful pregnancy outcomes but with a high rate of Caesarean section of 66 and 68% respectively. We therefore conducted a retrospective observational study of obstetric patients in a national spinal injuries centre from 1991 to 2016 with the aim of assessing pregnancy outcomes to allow accurate pre-conception and obstetric counselling of this high-risk group of women.

## Methods

We conducted a retrospective observational study where the inclusion criteria was any pregnant patient with spinal cord injury managed at Stoke Mandeville Hospital under the joint care of the obstetrics department and the National Spinal Injury Centre from 1991 to 2016 inclusively. Patients were identified from a hospital specific record of pregnant spinal cord injury patients referred to a named consultant obstetrician with responsibility for their care throughout the study period.

Maternity and hospital electronic and paper records were manually reviewed for demographic data, nature of spinal cord injury, level of disability, pre-pregnancy maternal comorbidities, antenatal complications, anaesthetic data, intrapartum data (including gestation at delivery, onset of labour, mode of delivery, indication for obstetric intervention) and neonatal outcomes. Data was collected using a standardised proforma and analysed using Microsoft Excel and Prism GraphPad software. Statistical analysis of demographic continuous variable data was performed using Shapiro-Wilk test of normality, with results expressed to reflect non-normal distribution.

## Funding

None.

## Results

Fifty women with a total of 68 pregnancies were identified and maternal demographic data are described in Table 1. Five women sustained SCI during pregnancy and the remaining 63 pregnancies were conceived at least 1 year after SCI, with the largest group of pregnancies (38%) occurring more than 10 years after the initial injury. The majority of SCI were caused by trauma (87%) and the level of disability was varied. The median patient body mass index (BMI) was 24 (inter-quartile range 21.7–33.3).

**Table 1:** Maternal demographic data

	Total	(%)	Cervical	T1 to T6	T7 to T10	T11 to T12	Lumbar
Number of Women	50		21	5	7	9	8
Number of Pregnancies	68		28	7	10	13	10
Time From Injury to Pregnancy (68 Pregnancies)							
Injured during Pregnancy	5	(7)	2	0	1	0	2
Less than 1 year	2	(3)	0	1	1	0	0
1 to 5 years	21	(31)	6	4	2	4	5
5–10 years	14	(21)	4	4	0	3	3
More than 10 years	26	(38)	10	5	2	1	8
Aetiology of Spinal Cord Injury							
Traumatic					44		(88)
Birth Trauma					2		(4)
Vascular					1		(2)
Infective					2		(4)
Transverse Myelitis					1		(2)
Bladder Management							
Normal					9		(18)
Intermittent self-catheterization					8		(16)
Indwelling urethral catheter					13		(26)
Suprapubic catheter					20		(40)

The most common antenatal complications in SCI patient were worsening of spasms (38%) and urinary tract infection (24%). There were no recorded hospital admissions antenatally for autonomic dysreflexia. Antenatal complications are described further in Table 2. The incidence of fetal malpresentation at term, including breech and transverse lie, was 13.6%, with a higher incidence in women with lesions above T6. External cephalic version (ECV) was undertaken in two women, with one successful and one failed.

**Table 2:** Antenatal outcomes

	Total	(%)	Cervical	T1 to T6	T7 to T10	T11 to T12	Lumbar
Number of Pregnancies	68		28	7	10	13	10
Live Births	66	(97)	27	7	10	13	9
Terminations	2	(3)	1	0	0	0	1
Primiparous	28	(41)	14	2	1	6	5
Multiparous	40	(59)	14	5	9	7	5
Fetal Presentation at Term (66 live births)							
Cephalic	57	(86)	21	7	10	10	9
Breech	8	(12)	5	0	0	3	0
Transverse	1	(1.5)	1	0	0	0	0
Antenatal Complications	No. of Women			(% of Total Number of Women *n* = 50)			
Worsening spasms	25			(38)			
Urinary Tract Infection	16			(24)			
PIH	3			(4.5)			
VTE (DVT/PE)	3			(4.5)			
Decubitus ulcers	2			(3)			

Intrapartum outcomes are described in Table 3. There were 66 live births, with two terminations for fetal abnormalities and no stillbirths. Vaginal delivery was achieved in 77% of pregnancies, including a 14% instrumental delivery rate, and 23% Caesarean delivery rate. All Caesarean sections were lower segment, of which 66.6% were elective and 33.3% were performed as emergencies for obstetric indications. All women with a lesion of T6 and above had regional anaesthesia during labour with a higher incidence of general anaesthesia for Caesarean delivery in women with lesions below T12 at 22% compared to 1.75% in women with lesions above T12. The rate of intrapartum autonomic dysreflexia was 6%.

**Table 3:** Intrapartum outcomes

	Total	(%)	Cervical	T1 to T6	T7 to T10	T11 to T12	Lumbar
Number of Live Births	66		27	7	10	13	9
Mode of birth							
SVD	42	(63)	12	7	8	8	7
OVD	9	(14)	9	0	0	0	0
EMCS	5	(8)	3	0	1	1	0
ELCS	10	(15)	3	0	1	4	2
Delivery							
Term	54	(82)	20	4	9	12	9
Preterm	12	(18)	7	3	1	1	0
Analgesia in Labour							
No regional anaesthetic	10	(15)	0	0	4	5	1
Epidural	21	(32)	13	4	1	1	2
Combined Epidural/Spinal	22	(33)	8	3	3	4	4
Spinal	10	(15)	6	0	2	2	0
General Anaesthetic	3	(5)	0	0	0	1	2
Onset of Labour							
Spontaneous	47	(71)	17	7	9	7	7
IOL	7	(11)	6	0	0	1	0
EMCS before Labour	2	(3)	1	0	0	1	0
ELCS before Labour	10	(15)	3	0	1	4	2
Indication for IOL							
PIH			2				
Prelabour ROM at term			1				
Meconium stained liquor			1				
Maternal discomfort			2				
IUGR			1				
Primary Indication for Caesarean			Elective	Emergency		Total	
Breech or transverse lie			4	1		5	
Cephalopelvic disproportion			2	0		2	
Previous TVT procedure			2	0		2	
Maternal Request			1	0		1	
Previous Caesarean			1	0		1	
Fetal distress			0	1		1	
Chorioamnionitis			0	1		1	
Failure to Progress			0	1		1	
Autonomic Dysreflexia			0	1		1	

Obstetric outcomes are described in Table 4. Preterm delivery at less than 37 weeks occurred in 18% of live births but only one at less than 34 weeks. There was a slightly higher incidence of preterm birth at 25% in women with lesions above T10 but there were no preterm deliveries in women with a lesion below T11 except for one extremely preterm delivery at 30 weeks by emergency Caesarean section for chorioamnionitis with severe maternal sepsis in a patient with a T12 lesion. There were two admissions to the neonatal unit – one for severe prematurity as described above and one for poor feeding in a small for gestational age baby with normal APGAR scores. All babies in this study had APGARS of 10 at 5 minutes. Mean birth weight was 3.15 kg (range 1.39 kg at 30 weeks to 3.9 kg). The incidence of small for gestational age defined as birthweight below the 10th centile by customized growth charts was 10%. There was one maternal death at 2 months postpartum following a vaginal delivery in a patient with a T12 lesion. This would be classified as a late indirect maternal death but she had been unwell with long term spinal injury complications both before and after pregnancy.

**Table 4:** Obstetric outcomes

Obstetric Outcomes	No. of births	(%)
Live Births	66	
Stillbirths	0	
Terminations	2	
Preterm Birth < 37 weeks	11	(15)
Preterm Birth < 34 weeks	1	(1.5)
Small for Gestational Age < 10th centile	7	(10)
Admission to NICU > 37 weeks	1	(1.5)
Admission to NICU < 37 weeks	1	(1.5)

## Discussion

Our results are consistent with other studies that show that pregnancies in women with spinal cord injury are high risk but pregnancy outcomes are generally good [10]. The obstetric management of women with SCI in our unit are summarised elsewhere and will not be repeated here except where relevant to the findings of the study [4].

We found a high rate of antenatal complications, particularly worsening of spasms (38%) which did not require any further treatment and urinary tract infections (24%). This is consistent with other studies suggesting a higher incidence of UTIs when compared with the general population [11] and supports the practice of frequent monitoring with consideration of antibiotic prophylaxis [6]. Fetal malpresentation at term, including breech and transverse lie, was more common with 13.6% incidence, approximately five times that of the general obstetric population at 3% [12], with a higher incidence of 21% in women with lesions above T6 affecting the abdominal myotomes. A possible mechanism for this increased rate of malpresentation could be the increased laxity of abdominal wall muscles in women with higher spinal cord injury. The primary indication for Caesarean section was malpresentation in five cases, with malpresentation co-existent in the other three cases where elective Caesarean was performed for alternative indications. In this study, we also report successful external cephalic version in one patient with a T11 injury with no adverse effects.

Women with SCI do not appear to be at higher risk of developing hypertensive disorders of pregnancy, with an incidence of 4.5% comparable to the national incidence of between 5 and 7% [13]. The incidence of venous thromboembolism (VTE) was higher at 4.5% than the background incidence in the general obstetric population of 0.2% however this result should be interpreted with caution as the three women in our study with proven VTE were known to have other pre-disposing risk factors such as SCI during pregnancy and high BMI of more than 50. Our practice is to perform standard VTE risk assessment in line with national guidance [14]. Pressure ulcers were less frequently encountered than expected as an antenatal complication occurring in 3% of pregnancies. Reported rates of pressure ulcers in the general spinal cord injury patient population are as high as 25 to 66% [15] and our findings may reflect pregnancy as a marker of good function and health.

Our study shows a prevalence of preterm birth at 18% which is higher than the estimated UK national rate of 5–9% [16] but lower than the reported rates in the literature regarding SCI patients of up to 27% [9]. Urinary tract infections are associated with an increased risk of preterm birth and in our study population, 33% of the women who had a preterm birth having also had a confirmed urinary tract infection during pregnancy. Women with lesions at and above T10 are at risk of not perceiving contractions and our data suggests that higher level spinal injuries are associated with a higher incidence of preterm labour of 25% in women with lesions above T10 and 4.5% in women with a lesion below T10. Reassuringly the only extremely preterm delivery at 30 weeks was iatrogenic for maternal urosepsis and the remainder occurred between 35 and 37 weeks of gestation.

Neonatal outcomes were good with a very low incidence of neonatal admission to NICU. The prevalence of small for gestational age babies below the 10th percentile by customised growth chart in our population was 10% which is comparable to the national incidence of 8% [17]. This is reassuring but the study is insufficiently large to detect any significant difference or to make any recommendations regarding monitoring of fetal growth and we would therefore recommend an individualised approach.

Our rate of Caesarean section at 23% is lower than reported in other studies of women with SCI, where rates of up to 69% are described [18]. This compares favourably with the Caesarean section rate in the general UK obstetric population which is reported as 27.1% in 2015–2016 [19]. The rate of Caesarean section in the general population has increased over the 25 year period covered by our study and this was mirrored in women with SCI but the relatively small numbers of patients delivering annually in our study cohort mean that further analysis of this trend is not possible. One qualitative study from Switzerland described some women with SCI reporting a lack of choice with regard to mode of delivery due to their medical providers’ lack of familiarity with their condition [20]. The rate of intrapartum autonomic dysreflexia was 6%. Of these four women, all had inadequate analgesia at the time of dysreflexia. Three had vaginal deliveries after successful regional anaethesia and one required an emergency Caesarean under spinal anaesthesia in order to achieve resolution of dysreflexia. We attribute this low rate of intrapartum autonomic dysreflexia to our protocol of early use of epidural anaesthesia in labour for women at risk of this complication.

All of the women over the 25-year period of our study were under the care of a designated consultant obstetrician, with an interest in spinal cord injury working at the same site as the National Spinal Injuries Centre, and specialised consultant, nursing, midwifery and anaesthetic staff familiar and confident in spinal cord injury management. Women received the majority of their antenatal care as outpatients in their local hospitals with late pregnancy and intrapartum management as inpatients in our hospital. We suggest that the relatively high numbers of SCI pregnancies managed in this unit may have contributed to this lower rate of Caesarean through experienced consistent antenatal and intrapartum management. The majority of women in this study successfully and safely achieved vaginal delivery regardless of the level of spinal cord injury and our data is reassuring in supporting patient choice with regard to mode of delivery.

This study reports pregnancy outcomes in a small population of high risk women with SCI and therefore it is difficult to generalise practice and derive meaningful comparisons between groups of women in this cohort however, this is the largest recent case series study since 1972 and may help counsel women regarding likely outcomes. Possible ascertainment bias is also a potential limitation given the retrospective nature of the study, however due to the hospital specific practice of referring all pregnant patients with spinal cord injury to a single designated obstetrician, it is unlikely that relevant cases were not included in the study. The length of the study over 25 years is a strength but may limit the generalizability of the outcomes, since some aspects of obstetric care, such as the rate of Caesarean section, have increased over time.

## Conclusion

Our data support the current evidence that spinal cord injury is high risk in pregnancy but that maternal and neonatal outcomes are generally successful and that vaginal delivery can be safely achieved in the majority of women, independent of the level of SCI.

## Data Availability

The raw datasets generated and analysed during the current study are not publicly available due to the potential for patient identification given the rare nature of the study population but are available from the corresponding author on reasonable request.

## Abbreviations

BMI: Body mass index
ECV: External cephalic version
SCI: Spinal cord injury
UTI: Urinary tract infection
VTE: Venous thromboembolism
